# Diabetes-related foot disease in Australia: a systematic review of the prevalence and incidence of risk factors, disease and amputation in Australian populations

**DOI:** 10.1186/s13047-021-00447-x

**Published:** 2021-01-19

**Authors:** Yuqi Zhang, Jaap J. van Netten, Mendel Baba, Qinglu Cheng, Rosana Pacella, Steven M. McPhail, Susanna Cramb, Peter A. Lazzarini

**Affiliations:** 1 Australian Centre for Health Services Innovation and Centre for Healthcare Translation, School of Public Health and Social Work, Queensland University of Technology, 60 Musk Ave, Kelvin Grove, Brisbane, QLD Australia; 2 Amsterdam UMC, University of Amsterdam, Department of Rehabilitation, Amsterdam Movement Sciences, Meibergdreef 9, Amsterdam, the Netherlands; 3 Podiatry Department, Sir Charles Gairdner Hospital, Perth, Australia; 4 The Kirby Institute, University of New South Wales, Sydney, Australia; 5 Institute for Lifecourse Development, University of Greenwich, London, UK; 6 Clinical Informatics Directorate, Metro South Health, Brisbane, Australia; 7 Allied Health Research Collaborative, The Prince Charles Hospital, Brisbane, Australia

**Keywords:** Diabetic foot, Diabetes complications, Diabetic neuropathies, Peripheral arterial disease, Foot ulcer, Amputation, Diabetes mellitus, Epidemiology

## Abstract

**Background:**

Diabetes-related foot disease (DFD) is a leading cause of global hospitalisation, amputation and disability burdens; yet, the epidemiology of the DFD burden is unclear in Australia. We aimed to systematically review the literature reporting the prevalence and incidence of risk factors for DFD (e.g. neuropathy, peripheral artery disease), of DFD (ulcers and infection), and of diabetes-related amputation (total, minor and major amputation) in Australian populations.

**Methods:**

We systematically searched PubMed and EMBASE databases for peer-reviewed articles published until December 31, 2019. We used search strings combining key terms for prevalence or incidence, DFD or amputation, and Australia. Search results were independently screened for eligibility by two investigators. Publications that reported prevalence or incidence of outcomes of interest in geographically defined Australian populations were eligible for inclusion. Included studies were independently assessed for methodological quality and key data were extracted by two investigators.

**Results:**

Twenty publications met eligibility and were included. There was high heterogeneity for populations investigated and methods used to identify outcomes. We found within diabetes populations, the prevalence of risk factors ranged from 10.0–58.8%, of DFD from 1.2–1.5%, and the incidence of diabetes-related amputation ranged from 5.2–7.2 per 1000 person-years. Additionally, the incidence of DFD-related hospitalisation ranged from 5.2–36.6 per 1000 person-years within diabetes populations. Furthermore, within inpatients with diabetes, we found the prevalence of risk factors ranged from 35.3–43.3%, DFD from 7.0–15.1% and amputation during hospitalisation from 1.4–5.8%.

**Conclusions:**

Our review suggests a similar risk factor prevalence, low but uncertain DFD prevalence, and high DFD-related hospitalisation and amputation incidence in Australia compared to international populations. These findings may suggest that a low proportion of people with risk factors develop DFD, however, it is also possible that there is an underestimation of DFD prevalence in Australia in the few limited studies, given the high incidence of hospitalisation and amputation because of DFD. Either way, studies of nationally representative populations using valid outcome measures are needed to verify these DFD-related findings and interpretations.

**Supplementary Information:**

The online version contains supplementary material available at 10.1186/s13047-021-00447-x.

## Background

Diabetes-related foot disease (DFD) is a leading cause of hospitalisation, amputation, disability and health care costs internationally [1–3]. DFD is typically defined as ulceration or infection of the foot associated with the key risk factors of peripheral neuropathy or peripheral artery disease (PAD) in people with diabetes [4]. Global estimates indicate approximately 130 million people have a key risk factor for DFD, 20 million of those have DFD [3], and up to 2 million of those require an amputation each year [1, 5].

Epidemiological research is the cornerstone of informing health care policy across the world [6]. Amputation incidence is frequently used in this regard as a key marker of the burden and health care quality of DFD [7, 8]. Recent large systematic reviews and meta-analyses have reported 4.6–4.8% of the global population with diabetes have foot disease [1, 9]. Australia was reported to have the lowest national foot disease prevalence with 1.5% [9]; but this Australian estimate was based on two studies whose data are now more than 15 years old, one of which only reported those with a history of DFD [10, 11]. Conversely, other reports published around the same time suggested Australia had one of the highest national diabetes-related amputation incidence rates amongst developed nations; but these findings came from non-peer reviewed government reports or narrative reviews [12, 13].

These collective findings of low DFD prevalence and high amputation incidence, suggests national health care delivery in Australia for people at risk of DFD may be effective to prevent DFD, but may not be as effective to prevent amputations in people that develop DFD. If these interpretations are correct, this would suggest that healthcare policymakers in Australia should focus more on treatment for those with DFD to prevent amputation and reduce high diabetes-amputation rates. Yet, these findings come from different studies, in different populations, with different risk factors, at different time periods, using different outcome measures; this makes interpretation challenging. Whilst one previous systematic review has reported on the epidemiology of chronic wounds in Australia that included foot ulcers as a wound type [14], no systematic review has comprehensively synthesised the population-based findings of risk factors for DFD, DFD and diabetes-related amputations. Thus, we aimed to systematically review the prevalence or incidence of risk factors for DFD (neuropathy, PAD, previous ulcer, previous amputation, foot deformity), of DFD (ulcers and infection), and of diabetes-related amputations (total, minor and major amputation) in Australia populations.

## Materials and methods

The systematic review was performed according to the Preferred Reporting Items for Systematic Review and Meta-Analyses (PRISMA) guidelines [15]. The review was prospectively registered in the PROSPERO database (CRD42016050740) and the protocol has been previously published [16]. We summarise the methods used below.

### Search strategy

The search included any original study published in any language until 31 December 2019 in the PubMed and EMBASE databases. These comprehensive databases were chosen as they cover all peer-reviewed publications back to 1951 (PubMed) and 1946 (EMBASE). We used search strings combining MeSH terms and keywords for prevalence or incidence, DFD or amputation, and Australia. The search strings were finalised after ensuring they identified a validation set of key publications known to the authors and are shown in Table S1 of the Supplementary material.

### Eligibility assessment

The title and abstract of each publication identified from the search were each screened by two investigators independently for eligibility (JJvN, YZ or MB). To be eligible for full-text assessment the publication had to report epidemiological data on risk factors for DFD, on DFD or on amputation in an Australian setting [16]. Any publication identified as eligible by at least one investigator was retrieved for full-text assessment. Cohen’s kappa was calculated for screening agreement between investigators.

Full-text assessments for each publication retrieved were also performed by two investigators independently for eligibility (YZ, JJvN, MB or QC) using the following inclusion and exclusion criteria. The inclusion criteria were that a publication needed to report an outcome of interest in a population of interest. The outcomes of interest for this review were risk factors for DFD (including neuropathy, PAD, previous ulcer, previous amputation or foot deformity), DFD itself (defined as “infection, ulceration, or destruction of tissues of the foot of a person with currently or previous diagnosed diabetes” [4]), and diabetes-related amputations (including total, minor or major amputation(s)) as defined by international guidelines [4, 16]. The populations of interest for this review were populations from a defined geographical catchment area of Australia, including general populations, community-dwelling populations with diabetes and inpatient populations. Any sub-groups of interest from those populations were also included, such as people with different types of diabetes (type 1 or 2 populations) and people of Aboriginal and Torres Strait Islander ethnicity (hereafter respectfully referred to as Indigenous populations). Exclusion criteria included publications that did not report original data, the geographical catchment population of the centre(s) concerned (unless data were standardised using Australian population demographics), or outcomes of interest that did not differentiate between people with and without diabetes [16]. There were no restrictions on study duration, study period and publication date. Investigators did not assess publications for which they were a (co-)author to prevent potential bias or conflict of interest. Disagreements in eligibility assessment between investigators were resolved by discussion between those investigators until consensus was reached, or if consensus was unable to be reached a third investigator decided. All publications deemed fully eligible by both investigators were those included in this systematic review.

### Quality assessment

Quality assessment of each included publication was also performed by two investigators independently (YZ, JJvN, MB, QC or PAL). Investigators did not assess publications for which they were a (co-)author to reduce potential bias or conflict of interest. A validated quality assessment tool designed to assess the risk of bias in population-based prevalence studies by Hoy and colleagues was used [17]. The tool assessed the quality of nine key methodological aspects of prevalence studies, including sample representation, random selection, acceptable case definitions, valid and reliable data collection instrument, and appropriate numerators and denominators [17]. Aspects specific to the content of this study included acceptable case definitions (if recommended in international guidelines definitions [4]), and valid and reliable data collection instrument (if the instrument or examination used had been previously reported as being valid and/or reliable). All items were afforded a score of one if reported and zero if not reported or unsure. The only exception to this was for the sample representation item, which scored two if the population of interest was a close representation of the national population, one if a close representation of a provincial (“state”) population within Australia, or zero if neither a national or state population (“regional”). The total quality score for each outcome was calculated as the sum of the nine assessment items, with the one item having a possible score of two, rendering the highest possible total score 10. The quality of the nine aspects was assessed at an outcome level rather than at a publication level. Thus, publications that reported multiple outcomes of interest had multiple total quality scores, one for each outcome reported. All disagreements in quality assessment between the two investigators were resolved by discussion until consensus was reached, or if consensus was unable to be reached a third investigator decided.

### Data extraction

Data were extracted into evidence tables from each included publication by one investigator (YZ, MB, or PAL), and then checked for accuracy by a second investigator (YZ, MB, or PAL). Investigators did not extract or check data from which they were a (co-)author to reduce potential bias or conflict of interest. The data extracted included study setting, design, period, population characteristics (including numbers, age, sex, diabetes type, ethnic groups), outcomes of interest (including method definitions used by the publication to identify the outcome), use of prevalence or incidence, outcome number, and proportion or rate. If the same original findings were reported from the same study in two or more publications, we used the findings from the earlier publication. However, if additional numbers or findings were reported we used the later publication. Disagreements in data extraction between investigators were resolved by discussion until consensus was reached, or if consensus was unable to be reached a third investigator decided.

### Data analysis

The summary measure used for each outcome of interest in each included publication was proportion for prevalence (%) or rate for incidence (per person-years). If a publication only reported the outcome number, the number was converted to a prevalence proportion, or crude incidence rate, by dividing the outcome of interest number (numerator) by the population of interest number (denominator).

Eligibility criteria for performing a meta-analysis included that there were at least three publications reporting on the same outcome of interest, using similar definitions to identify the outcome, in a similar population of interest, with data collected within 10 years of each other. Meta-analyses would be used to calculate pooled incidence or prevalence estimates using a random-effects model, with the *I*^2^ test used to examine heterogeneity across publications.

No investigations of the effects of the risk of bias were performed, including publication bias and selective reporting within studies, as epidemiological studies rarely prospectively register protocols and as such unpublished results to calculate publication bias could not be estimated. Therefore, the confidence in the cumulative evidence was a descriptive analysis based on the quality assessment of the included publications.

## Results

**Fig. 1:**
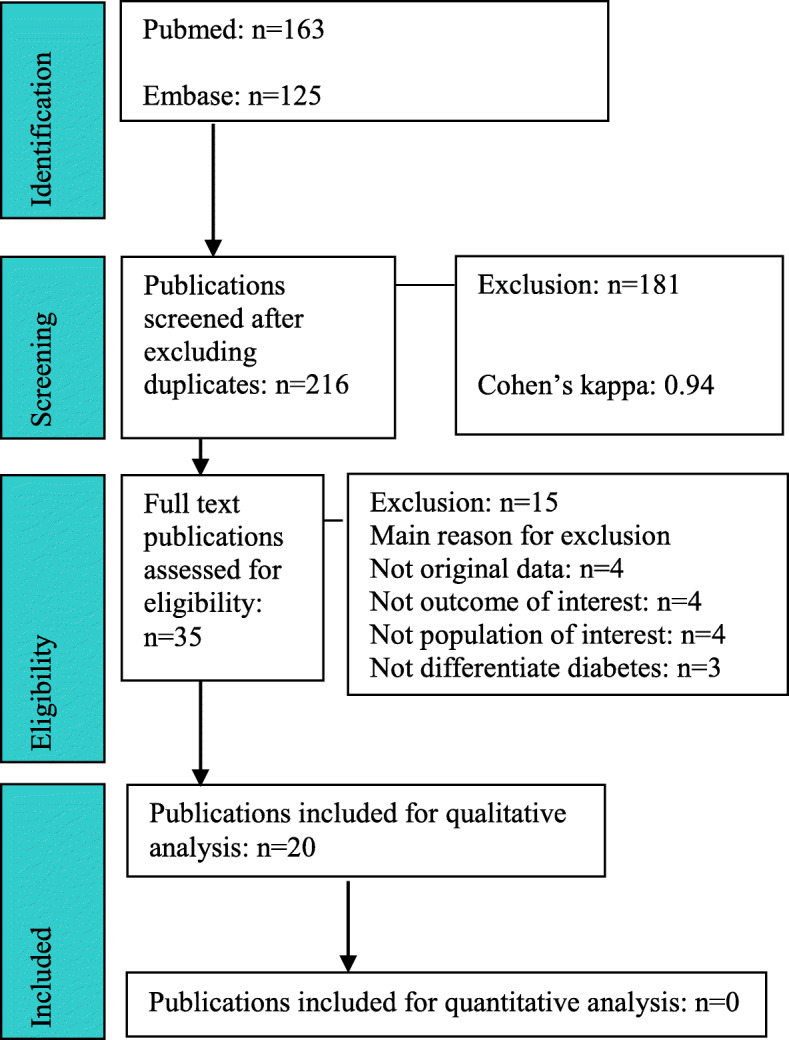
Preferred Reporting Items for Systematic Reviews and Meta-Analyses flow diagram

A total of 216 unique publications were identified from the original search. After title and abstract screening, 181 publications were excluded with 35 remaining for full-text assessment. Screening agreement between investigators was very high (Cohen’s kappa: 0.94). After full-text assessment, 20 publications [10, 11, 18–35] were included in this systematic review (see Fig. 1) with 15 excluded for not meeting inclusion criteria (see Table S2). The eligibility criteria for performing any meta-analysis was not met for any outcome of interest and therefore only qualitative analyses of included publications are reported.

### Data extraction

A summary of the characteristics of each of the 20 included publications is provided in Table 1. The settings reported included 15% (three of 20) that were nation-wide [10, 20, 31]; 45% state-wide (including six from Queensland (Qld), two from Western Australia (WA) and one from Victoria (Vic)) [21, 23–29, 33]; and 40% were region-wide settings (including five from Fremantle (WA) and one each from Far North Qld (Qld), Darwin (Northern Territory (NT)) and Central Australia) [11, 18, 19, 22, 30, 32, 34, 35]. The study designs included 35% that were prospective [11, 18, 19, 29, 32, 34, 35], 20% were cross-sectional [10, 25–27], and the remaining 45% were of retrospective design [20–24, 28, 30, 31, 33]. The primary populations of interest investigated included 25% that were a general population, 50% a community-dwelling population with diabetes, and 25% an inpatient population. Subpopulations were only reported amongst publications investigating community-dwelling populations with diabetes and included six publications reporting type 2 diabetes and two each for type 1 diabetes and Indigenous populations. The outcomes of interest reported included eight publications reporting one or more risk factors for DFD, five reporting DFD, eleven reporting diabetes-related amputation, and four reporting aggregated outcomes that included different combinations of risk factors or DFD. Table 2 displays a summary of the prevalence and incidence findings for the outcomes of interest. Additionally, the evidence tables for each outcome of interest are presented in the Supplementary Material, including Table S3 for risk factors of DFD, Table S4 for DFD, Table S5 for diabetes-related amputations and Table S6 for aggregated risk factors or DFD outcomes.

**Table 1:** Summary characteristics of the 20 included publications

Reference (***alphabetical order***)	Setting	Study Design	Period	Population(s) of interest	Population numbers (n)	Subpopulation investigated	Outcome(s) reported	QA total score^a^
Baba,2014 [18]	Region-wide: Fremantle, WA	Prospective cohort study	1993–1996	Community-dwelling population with type 2 diabetes	*n* = 1292	Type 2	PN PAD Foot ulcer Foot ulcer hospitalisation	7 7 5 5
Baba,2015a [35]	Region-wide: Fremantle, WA	Prospective cohort study	1993–1996; 2008–2011	Community-dwelling population with type 2 diabetes	*n* = 1509	Type 2	PN PAD Previous foot ulcer Amputation	7 7 5 5
Baba,2015b [34]	Region-wide: Fremantle, WA	Prospective cohort study	1993–1996; 2008–2011	Community-dwelling population with type 2 diabetes	*n* = 1296; n = 1509	Type 2	Foot ulcer	5
Clarke,2006 [33]	State-wide: Qld	Retrospective study	1995–1999	Community-dwelling population with diabetes	*n* = 20,538	–	Amputation	7
Commons,2015 [19]	Region-wide: Darwin, NT	Prospective cohort study	2012–2013	General population	*n* = 192,680	–	Diabetic foot infection hospitalisation Amputation	7 7
Davis,2012 [32]	Region-wide: Fremantle, WA	Prospective cohort study	1993–1996; 2008–2011	Community-dwelling population with type 2 diabetes	n = 1296; n = 1509	Indigenous; non-Indigenous	PN PAD	7 7
Davis,2006 [11]	Region-wide: Fremantle, WA	Prospective cohort study	1993–1996	Community-dwelling population with type 2 diabetes	*n* = 1294	Type 2	Amputation	5
Dillon,2017 [31]	Nation-wide: Australia	Retrospective cohort study	2007–2012	General population	NS	Type 1 Type 2	Amputation	8
Ewald,2001 [30]	Region-wide: Central Australia	Retrospective cohort study	1992–1997	Inpatient population with diabetes	NS	–	Foot complication	5
Jia,2017 [29]	State-wide: Qld	Prospective cohort study	2012–2014	Community-dwelling population with diabetes and an uninfected foot ulcer	*n* = 853	–	PN PAD Previous foot ulcer Previous amputation Foot deformity Foot infection	9 9 8 8 9 9
Kurowski,2015 [28]	State-wide: WA	Retrospective study	2000–2010	Community-dwelling population with diabetes	NS	Type 1 Type 2	Amputation	7
Lazzarini,2015 [24]	State-wide: Qld	Retrospective study	2005–2010	General population Community-dwelling population with diabetes	*n* = 24,990,524 *n* = 846,967	–	Foot complication hospitalisation Amputation	7 7
Lazzarini,2016a [27]	State-wide: Qld	Cross-sectional study	2013	Inpatient population Inpatient population with diabetes	*n* = 733 *n* = 172	–	Foot ulcer Foot infection Foot complication hospitalisation	8 8 7
Lazzarini,2016b [26]	As above	As above	As above	As above	As above	–	Critical PAD Amputation	8 8
Lazzarini,2017 [25]	As above	As above	As above	As above	As above	–	PN PAD Previous foot ulcer Previous amputation Foot deformity Foot complications	7 7 7 7 7 7
Norman,2010 [23]	State-wide: WA	Retrospective study	2000–2008	Community-dwelling population with diabetes	NS	Indigenous; non-Indigenous	Amputation	6
O’Hara,1998 [21]	State-wide: Vic	Retrospective study	1993–1995	Inpatient population with diabetes	*n* = 95,091	–	PAD Amputation	6 6
O’Rourke,2012 [22]	Region-wide: Far North Qld	Retrospective study	1999–2008	General population	*N* = 262,000	–	Major amputation	6
Payne,2000 [20]	Nation-wide: Australia	Retrospective study	1995–1998	General population	NS	–	Amputation	8
Tapp,2003 [10]	Nation-wide: Australia	Cross-sectional study	1999–2000	Community-dwelling population with diabetes	*n* = 821	–	PN PAD Previous foot ulcer	9 9 7

^a^QA total scores listed correspond to, and are in the same sequence to, the outcomes reported in each publication. *NS* not stated; *NT* Northern Territory; *PAD* peripheral artery disease; *PN* peripheral neuropathy; *QA* quality assessment; *Qld* Queensland; *Vic* Victoria; *WA* Western Australia

**Table 2:** Summary of the prevalence and incidence findings for the outcomes of interest

Summary results by population	Risk factors						DFD		DFD-related amputation		
	Peripheral Neuropathy [10, 18, 25, 29, 32, 35]	PAD [10, 18, 21, 25, 26, 29, 32, 35]	Previous foot ulcer [10, 25, 29, 35]	Previous amputation [25, 29]	Foot deformity [27, 29]		Foot ulcer [18, 27, 34, 35]	Foot infection [19, 27, 29]	Total [11, 20, 21, 24, 26, 28, 31, 33]	Minor [11, 19, 23, 24, 28]	Major [11, 19, 22–24, 28]
**Prevalence**											
General population	–	–	–	–	–		–	–	–	–	–
Community dwelling diabetes population^a^	10.0–58.2%	10.3–29.2%	0.5–2.1%	–	–		1.2–1.5%	–	–	–	–
Inpatient diabetes population^a^	43.3%	35.3%	20.3%	9.3%	30.5%		15.1%	7.0%	1.4–5.8%	–	–
**Incidence**											
General population	–	–	–	–	–	–		79 hospitalisations /100,000	14.0–17.8 /100,000	12.0–24.0 /100,000	5.8–9.3 /100,000
Community dwelling diabetes population^a^	–	–	–	–	–	–	5.2–36.6^ hospitalisations /1000	–	5.2–7.2 /1000	3.5–4.8 /1000	1.7–2.4 /1000
Inpatient diabetes population^a^	–	–	–	–	–	–	–	–	–	–	–

^a^This population contains a range from outcomes in populations that included all diabetes, type 2 and/or type 1 diabetes. ^This range also includes aggregated DFD outcome hospitalisations

*DFD* diabetic foot disease; *DFU* diabetic foot ulcer; *NS* not stated; *NT* Northern Territory; *PAD* peripheral artery disease; *PY* person years; *Qld* Queensland; *Vic* Victoria; *WA* Western Australia

### Quality assessments

Quality assessments were performed for 45 outcomes reported across the 20 included publications as displayed in Table 3. The median total quality score (IQR) was 7 (7-8) and scores ranged from 5 to 9 (from a total possible score of 10). Items recording higher risk of bias scores included population of interest not representative of a national population (89%, 40 of 45 outcomes; with 36% also not representative of a state population), outcome of interest not measured using a valid or reliable data collection instrument (60%), not collecting data directly from participants (38%), having a non-response bias (27%), and not using a random selection or census method to identify the population of interest (24%).

**Table 3:** Final quality assessment scores at an outcome level for all included publications

Reference (alphabetical order)	Foot Outcome	Quality assessment (Hoy,2012)^a^									
		1. Representation of national population^b^	2. True representation	3. Random Selection	4. Minimal Non-response bias	5. Data collected directly	6. Acceptable Case Definition	7. Data collection instrument valid and reliable ^c^	8. Data collection mode consistent	9. Numerator(s) and denominator(s) appropriate	Total Score
Baba,2014 [18]^d^	PN	0	1	1	0	1	1	1	1	1	7
	PAD	0	1	1	0	1	1	1	1	1	7
	Foot ulcer	0	1	1	0	1	0	0	1	1	5
	Foot ulcer hospitalisation	0	1	1	0	0	1	0	1	1	5
Baba,2015a [34]^d^	PN	0	1	1	0	1	1	1	1	1	7
	PAD	0	1	1	0	1	1	1	1	1	7
	Previous foot ulcer	0	1	1	0	0	1	0	1	1	5
	Amputation	0	1	1	0	1	1	0	1	1	5
Baba,2015b [35]^d^	Foot ulcer	0	1	1	0	1	0	0	1	1	5
Clarke,2006 [33]	Amputation	1	1	1	1	0	1	0	1	1	7
Commons,2015 [19]^d^	Diabetic foot infection hospitalisation	0	1	1	1	1	1	1	1	0	7
	Amputation	0	1	1	1	1	1	1	1	0	7
Davis,2012 [32]^d^	PN	0	1	1	0	1	1	1	1	1	7
	PAD	0	1	1	0	1	1	1	1	1	7
Davis,2006 [11]	Amputation	0	1	1	0	0	1	0	1	1	5
Dillon,2017 [31]	Amputation	2	1	1	1	0	1	0	1	1	8
Ewald,2001 [30]	Foot complication	0	0	1	1	0	1	0	1	1	5
Jia,2017 [29]^d^	PN	1	1	1	1	1	1	1	1	1	9
	PAD	1	1	1	1	1	1	1	1	1	9
	Previous foot ulcer	1	1	1	1	1	1	0	1	1	8
	Previous amputation	1	1	1	1	1	1	0	1	1	8
	Foot deformity	1	1	1	1	1	1	1	1	1	9
	Foot infection	1	1	1	1	1	1	1	1	1	9
Kurowski,2015 [28]	Amputation	1	1	1	1	0	1	0	1	1	7
Lazzarini,2015 [24]^d^	Hospitalisation for foot complication	1	1	1	1	0	1	0	1	1	7
	Amputation	1	1	1	1	0	1	0	1	1	7
Lazzarini,2016a [26]^d^	Foot ulcer	1	1	0	1	1	1	1	1	1	8
	Foot infection	1	1	0	1	1	1	1	1	1	8
	Foot complication hospitalisation	1	1	0	1	1	1	0	1	1	7
Lazzarini,2016b [27]^d^	Critical PAD	1	1	0	1	1	1	1	1	1	8
	Amputation	1	1	0	1	1	1	1	1	1	8
Lazzarini,2017 [25]^d^	PN	1	1	0	1	1	1	0	1	1	7
	PAD	1	1	0	1	1	1	0	1	1	7
	Previous foot ulcer	1	1	0	1	1	1	0	1	1	7
	Previous amputation	1	1	0	1	1	1	0	1	1	7
	Foot deformity	1	1	0	1	1	1	0	1	1	7
	Foot complications	1	1	0	1	1	1	0	1	1	7
Norman,2010 [23]	Amputation	1	1	1	1	0	0	0	1	1	6
O’Hara,1998 [21]^d^	PAD	1	1	1	1	0	0	0	1	0	6
	Amputation	1	1	1	1	0	0	0	1	0	6
O’Rourke,2012 [22]	Major amputation	0	1	1	1	0	1	0	1	1	6
Payne,2000 [20]	Amputation	2	1	1	1	0	1	0	1	1	8
Tapp,2003 [10]^d^	PN	2	1	1	1	0	1	1	1	1	9
	PAD	2	1	1	1	0	1	1	1	1	9
	Previous foot ulcer	2	1	1	1	0	0	0	1	1	7
TOTAL	45 publication-outcomes	5 (24)^e^	44	34	33	28	39	18	45	41	

^a^ All items were scored 1 = Low risk of bias or 0 = High risk of bias

^b^This item was scored: 2 = If the population of interest was a close representation of the national population; 1 = If the population of interest was a close representation of a provincial (state) population; 0 = If the population of interest was neither a close representation of a national or provincial population

^c^ Data collection instrument was considered as valid and reliable if the instrument or examination used had been previously reported as being valid and/or reliable;

^d^ The same publication reported several outcomes of interest that were individually quality assessed

^e^ Indicates five recorded a score of 2 (close representation of a national population) and 24 as score of 1 (close representation of a provincial/regional population)

### Risk factors

Eight publications [10, 18, 21, 25, 27, 29, 32, 35] reported the prevalence of different risk factors for DFD and none reported incidence (Table 2; Table S3).

### Peripheral neuropathy

Six publications [10, 18, 25, 29, 32, 35] reported neuropathy prevalence, with all using acceptable case definitions and validated clinical examination methods for measuring the outcome of neuropathy. This included four publications investigating neuropathy within a community-dwelling diabetes population, one in a community-dwelling DFD population and one in an inpatient population. In the community-dwelling diabetes populations, neuropathy prevalence was 10.0% in a nation-wide population, and 30.8–58.2% in region-wide Fremantle populations (including 38.9–48.5% in Indigenous and 33.6–63.3% in non-Indigenous populations) [10, 18, 32, 35]. In a state-wide Qld community-dwelling DFD population, neuropathy prevalence was 85.0% [29]. In a state-wide Qld inpatient population, diabetes-related neuropathy prevalence was 10.2% (including 43.3% in diabetes inpatients) [25].

### Peripheral artery disease (PAD)

Eight publications [10, 18, 21, 25, 27, 29, 32, 35] reported PAD prevalence, with all using acceptable case definitions and validated clinical examination methods for measuring PAD, except one that used unvalidated hospital coding methods. This included four investigating PAD in a community-dwelling diabetes population, one a community-dwelling DFD population and three in an inpatient population. In the community-dwelling diabetes populations, PAD prevalence was 10.3% in a nation-wide population, 2.7% in state-wide Vic population (hospital coding) [21], and 22.6–29.2% in region-wide Fremantle population (including 15.8–30.7% in Indigenous and 21.5–29.7% in non-Indigenous populations) [18, 32, 35]. In a state-wide Qld community-dwelling DFD population, PAD prevalence was 45.8% [29]. In a state-wide Qld inpatient population, diabetes-related PAD prevalence was 8.2% (including 35.1% in diabetes inpatients) [25, 27].

### Previous foot ulcer

Four publications [10, 25, 29, 35] reported previous foot ulcer prevalence, with all measured using acceptable case definitions but unvalidated self-report methods, except one that did not report an acceptable definition [10]. This included two investigating for those with previous (healed) foot ulcers in a community-dwelling diabetes population [10, 35], one in a community-dwelling DFD population [29] and one in an inpatient population [25]. In the community-dwelling diabetes populations, previous foot ulcer prevalence was 2.1% in a nation-wide population [10], and 0.5–1.8% in region-wide Fremantle (WA) populations [35]. In a state-wide Qld community-dwelling DFD population, previous foot ulcer prevalence was 69.8% [29]. In a state-wide Qld inpatient population, diabetes-related previous foot ulcer prevalence was 4.8% (including 20.3% in diabetes inpatients) [25].

### Previous amputation

Two publications [25, 29] reported previous amputation prevalence, with both using an acceptable case definition but unvalidated clinical examination method, including one investigating those with previous (healed) amputations in a community-dwelling DFD population [29] and one in an inpatient population [25]. In a state-wide Qld community-dwelling DFD population, previous amputation prevalence was 28.4% [29]. In a state-wide Qld inpatient population, diabetes-related previous amputation prevalence was 2.2% (including 9.3% in diabetes inpatients) [25].

### Foot deformity

Two publications [25, 29] reported foot deformity prevalence, with both using an acceptable case definition but unvalidated clinical examination methods, including one investigating in a community-dwelling DFD population and the other an inpatient population. In a state-wide Qld community-dwelling DFD population, foot deformity prevalence was 63.2% [29]. In a state-wide Qld inpatient population, diabetes-related foot deformity prevalence was 7.2% (including 30.5% in diabetes inpatients) [25].

### Diabetes-related foot disease (DFD)

Five publications [18, 19, 27, 29, 35] reported the outcomes of prevalence or incidence of DFD (Table 2; Table S4).

### Foot ulcer

#### Prevalence

Three publications [18, 27, 35] reported foot ulcer prevalence, with only one [27] using an acceptable case definition and validated clinical examination method. Two were investigating foot ulcers in a community-dwelling diabetes population and one in an inpatient population. In the community-dwelling diabetes populations, foot ulcer prevalence was 1.2–1.5% in the region-wide Fremantle population [18, 35]. In a state-wide Qld inpatient population, diabetes-related foot ulcer prevalence was 3.5% (including 15.1% in diabetes inpatients) [27].

#### Incidence

One publication [18] reported foot ulcer hospitalisation incidence using an acceptable case definition but unvalidated hospital coding method (Table S4). The publication reported an incidence of 5.2 per 1000 person-years in a region-wide Fremantle community-dwelling diabetes population.

### Foot infection

#### Prevalence

One publication [27] reported foot infection prevalence, using an acceptable case definition and validated clinical examination method in an inpatient population. In a state-wide Qld inpatient population, diabetes-related foot infection prevalence was 1.7% (including 7.0% in diabetes inpatients) [27].

#### Incidence

Two publications [19, 29] reported foot infection incidence, with both using an acceptable case definition and validated clinical examination method, including one in a general population and one in a community-dwelling DFD population (Table S4). In a region-wide Darwin general population, the foot infection hospitalisation incidence was 79 per 100,000 person-years (including 195 per 100,000 in Indigenous and 38 per 100,000 all person-years in non-Indigenous populations) [19]. In a state-wide Qld community-dwelling population, 40.1% of people with non-infected diabetes-related foot ulcers developed an infection over a 12 month period [29].

### Diabetes-related amputation

Eleven publications [11, 19–24, 26, 28, 31, 33] reported the outcomes of prevalence or incidence of diabetes-related amputations (Table 2; Table S5).

### Total amputations

#### Prevalence

Two publications [21, 26] reported total amputation procedure prevalence, with only one using an acceptable case definition and validated clinical examination method [26]. In a state-wide Qld inpatient population, diabetes-related amputation procedure prevalence was 1.4% (clinical methods) (including 5.8% in diabetes inpatients) [26]. In a state-wide Vic inpatient population with diabetes, amputation procedure prevalence was 1.4% (hospital coding) [21].

#### Incidence

Six publications [11, 20, 24, 28, 31, 33] reported total amputation procedure incidence, with all using an acceptable case definition and unvalidated hospital coding method, except one using a validated clinical examination method [24]. This included two investigating amputations procedures in a general population [20, 31], three in a community-dwelling diabetes population [11, 28, 33], and one reporting in both populations [24]. In the general populations, diabetes-related total amputation incidence was 14.0–16.5 per 100,000 person-years in nation-wide populations [20, 31], and 17.8 per 100,000 in a state-wide Qld population [24]. In the community-dwelling diabetes population, total amputation incidence was 5.2–6.9 per 1000 person-years in a state-wide Qld diabetes populations [24, 33], 5.6 (type 2) and 7.2 (type 1) per 1000 in a state-wide WA diabetes population [28], and 6.0 per 1000 (type 2) in a region-wide Fremantle population (including 3.8 per 1000 for first ever amputation procedure) [11].

### Minor amputations

Five publications [11, 19, 23, 24, 28] reported minor amputation procedure incidence, with all using an acceptable case definition and unvalidated hospital coding method [11, 23, 24, 28], except one using a validated clinical examination method [19]. This included two investigating amputation procedures in a general population [19, 23], two in a community-dwelling diabetes population [11, 23, 28], and one reporting both [24]. In the general populations, diabetes-related minor amputation incidence was 12.0 per 100,000 person-years in a state-wide Qld population [24], 24.0 per 100,000 in a region-wide Darwin population (clinical method) [19], and 28.9 per 100,000 in a state-wide WA non-Indigenous population (≥50 years of age) and 185.0 in an Indigenous population (≥50 years of age) [23]. In the community-dwelling diabetes populations, minor amputation incidence was 3.5 per 1000 person years in a state-wide Qld diabetes population [24], and 3.9 (type 2) and 4.8 (type 1) per 1000 in a state-wide WA diabetes population [28], and 2.3 for first ever minor amputation procedure per 1000 (type 2) in a region-wide Fremantle diabetes population [11].

### Major amputations

Six publications [11, 19, 22–24, 28] reported major amputation incidence, again with all using an acceptable case definition and unvalidated hospital coding methods, except one using a validated clinical examination method [19]. This included three in a general population [19, 22, 23], two in a community-dwelling diabetes population [11, 23, 28], and one reporting both [24]. In the general populations, diabetes-related major amputation incidence was 5.8 per 100,000 person-years in a state-wide Qld population [24], 7.6 per 100,000 region-wide NT (Darwin) population (clinical method) [19], 9.3 per 100,000 in region-wide Far North Qld population [22], and 13.1 (> 50 years non-Indigenous) and 76.8 (> 50 years Indigenous) per 100,000 in state-wide WA population [23]. In the community-dwelling diabetes populations, major amputation incidence was 1.7 per 1000 person-years in a state-wide Qld diabetes population [24], 1.8 and 2.4 per 1000 (type 2 and 1 respectively) in a state-wide WA diabetes population [28], and 1.8 for first ever major amputation procedure per 1000 (type 2) in a region-wide Fremantle diabetes population [11].

### Aggregated risk factors or DFD outcomes

Four publications [24, 25, 27, 30] also reported the prevalence or incidence of aggregated outcomes that included different combinations of risk factors or DFD outcomes (Table S6). All used acceptable case definitions for the aggregated outcomes but unvalidated clinical examination or hospital coding methods. This included one in a general population [30], one in a community-dwelling diabetes population [24] and two in an inpatient population [25, 27]. In the general population, incidence of hospitalisation for an aggregated DFD-related outcome (neuropathy, PAD, foot ulcer, foot infection, and/or amputation) was 98–285 per 100,000 person years in a Central Australia general population over 15 years of age during 1992–1997 [30]. In a state-wide Qld community-dwelling population with diabetes, incidence of hospitalisation for an aggregated DFD-related outcome (neuropathy, PAD, foot ulcer, foot infection, amputation) was 20.2–36.6 per 1000 person-years during 2005–2010 [24]. In a state-wide Qld inpatient population with diabetes, the prevalence of being hospitalised for the primary reason of an aggregated DFD-related outcome (neuropathy, PAD, foot deformity, previous foot ulcers, previous amputation, foot ulcer, infection, amputation) was 8.7% and the prevalence of having at least one risk factor, DFD or amputation outcome present (neuropathy, PAD, foot deformity, previous foot ulcers, previous amputation) was 65.5% [25, 27].

## Discussion

We systematically reviewed the prevalence and incidence of risk factors for DFD, DFD itself and diabetes-related amputations in Australia. We found 20 publications that reported on 45 outcomes within geographically defined populations of Australia. Within community-dwelling populations with diabetes in Australia, the prevalence of those with key risk factors ranged from 10.0–58.2%, those with DFD from 1.2–1.5%, and the incidence of diabetes-related amputation ranged from 5.2–7.2 per 1000 person-years. We also found the incidence of those with diabetes hospitalised for DFD-related outcomes ranged from 5.2–36.6 per 1000 person-years. Furthermore, within those hospitalised with diabetes, the prevalence of key risk factors ranged from 35.3–43.3%, DFD from 7.0–15.1% and those having amputation procedures during their hospitalisation from 1.4–5.8%. However, there was a high level of heterogeneity between studies for the populations investigated and the quality of methods used to measure outcomes.

Our synthesised findings suggest that within people with diabetes in Australia there is a relatively high proportion that have risk factors for developing DFD, while only a low proportion develop DFD, and a high proportion of these appear to be hospitalised for DFD or undergo amputations. However, the quantity and quality of findings varied between outcomes. We found many more publications reported on risk factors (eight publications) or diabetes-related amputations outcomes (eleven), than on DFD itself (five). Furthermore, most publications reporting risk factors or amputations used acceptable methods to measure outcomes within a broad range of different populations; whereas, most publications reporting DFD did not use acceptable methods of measurement and most were limited to within regional populations. Therefore, we are more confident that our synthesised findings indicating a high prevalence of risk factors and high incidence of diabetes-related amputations are an accurate reflection of the burden of the Australian population, than we are of a low prevalence of DFD in Australia. With those caveats in mind, it is useful to compare our synthesised Australian findings to similar global findings to help interpret the national burden and health care quality of DFD in Australia.

### Risk factors

For key risk factors in community-dwelling populations with diabetes, our Australian findings were somewhat similar to those from global reviews [1, 36–39]. The 10–58% range for neuropathy prevalence compared closely with a 10–50% range [37] and a 34% pooled global prevalence estimate [1] reported in two recent global reviews. The 10–29% PAD prevalence range, and 35% inpatient prevalence, also compared closely with a 20–29% range identified in a global review of diabetes populations > 40 years [36], and with a 29–36% range in inpatients [38, 39]. This suggests the national burden of key risk factors for DFD, and perhaps the quality of care for people with diabetes to prevent key risk factors for DFD in Australia, is on par with other countries where data are available.

Interestingly, we found no marked differences between Indigenous and non-Indigenous people for these risk factors, in contrast to a previous systematic review [40]. This may be explained by our tighter inclusion criteria that included only population-based publications and in turn only identified studies from urban settings [32], while the previous review predominantly identified single site clinic-based studies from remote settings [40], with geographical remoteness known to increase DFD rates [41].

### Diabetes-related foot disease

In community-dwelling populations with diabetes, we identified a DFU prevalence range of 1.2–1.5%. In 2020, this would equate to ~ 16,400–20,500 Australians having DFD (based on 1.2–1.5% of the 1.37 million Australians with diabetes) [18, 34]. These findings are much lower than the two recent pooled global prevalence estimates of 4.6 and 4.8% [1, 9], but similar to the 1.5% pooled Australian estimate from the same global review [9]. Interestingly though, for inpatient populations with diabetes, we identified a DFD prevalence range of 7.0–15.1% [26, 27] which was much higher than a recent pooled global prevalence estimate of 7.1% [9]. This suggests that preventing DFD in Australia may be more effective than in other countries, but care for those developing DFD may be less effective.

These interpretations are supported by our aggregated DFD-related hospitalisation incidence findings. We found 5.2–36.6 per 1000 person-years with diabetes, which would equate to ~ 7100–50,000 DFD-related hospitalisations each year in Australia [42]. Based on our earlier estimates that ~ 16,400–20,500 Australians have DFD, this would suggest there are ~ 0.3–3.1 hospitalisations for each Australian with DFD each year. However, as identified earlier, we are less confident of the accuracy of our low DFD prevalence findings. This lack of confidence is perhaps emphasised by a recently reported adjusted estimate that ~ 45,000 Australians have DFD [1]. This estimate would equate to a ~ 3.2% DFD prevalence in Australia which is much higher than the 1.2–1.5% prevalence finding from this review [1]. This recent adjusted estimate was based on a validated Bayesian meta-regression model that used similar Australian literature to our review, but also adjusted for multiple known factors that influence DFD prevalence such as national obesity prevalence and income per capita, and is therefore perhaps a more realistic estimate of the Australian DFD prevalence [1].

Overall, these collective DFD findings suggest that perhaps the DFD prevalence findings in our review are a considerable under-estimate of the reality of the Australian national DFD burden [1]. Thus, large epidemiology studies investigating DFD using validated methods in nationally representative populations are needed to shed more light on the reality of the national DFD burden. Yet, with more confidence in our estimates that ~ 7100–50,000 DFD-related hospitalisations are occurring each year in Australia, urgent improvements are needed in the understanding and delivery of national treatment services for people with DFD to reduce such a large national hospitalisation burden.

### Diabetes-related amputation

We also found a comparatively high 5.2–7.2 total amputation incidence range per 1000 person-years with diabetes in Australia, compared with the 1.4–7.0 range reported in a recent global systematic review [43]. Similarly, we found what appears to be a very high 14.0–17.8 total amputation incidence range per 100,000 person-years in the general population, compared with a recent median rate over time of 9.4–9.9 reported for 26 Organization for Economic Cooperation and Development (OECD) nations [44]. Whilst, the OECD median rate excluded toe amputation procedures and those < 15 years of age, when interpreted with the diabetes population findings these Australian total diabetes-related amputation rates still seem comparatively high.

For a more comprehensive interpretation of amputation, major and minor amputation rates should be teased out [7, 45]. Whilst major amputations are a devastating final resort treatment often performed to preserve life in those with severe DFD, minor amputations can be considered to preserve the limb in those with moderate-to-severe DFD, and have much different effects on quality of life [7, 45]. We found a 1.7–2.4 major amputation incidence range per 1000 person-years with diabetes in Australia, and well within the range of 0.3–3.8 reported in the recent global systematic review [43]. However, the 3.5–4.8 range for minor amputations was much higher than the 0.9–3.6 reported in the global review [43]. This again seems to support our earlier interpretation that national treatment delivery in the community for people with DFD may not be effective in preventing hospitalisation, resulting in more people hospitalised for DFD receiving more minor amputation than in other nations.

We also found differences in amputation rates within different sub-populations of Australia. Interestingly, we found up to 38 fold higher rates for amputations in Indigenous populations compared to the non-Indigenous population [23], and much higher than the 3–6 fold higher rates for amputations in Indigenous populations reported in the previously discussed systematic review [40]. Perhaps implicated in this finding, was that we also found geographical variation in amputation rates in two nation-wide publications [20, 31]. These studies found higher diabetes-related amputation rates in the most geographically remote state/territory of the Northern Territory than all other states [20, 31]. Furthermore, two statewide studies found significant decreases in diabetes-related amputation rates over time following improved statewide health care delivery for people with DFD [24, 28]. These collective findings again point to higher amputation rates in those (sub-)populations with lower access to DFD treatment, but these rates may be reducible when access to DFD health care treatment is improved in Australia. Lastly, we found those with type 1 diabetes had slightly higher rates than those with type 2 diabetes (7.2 total amputations per 1000 person-years in type 1 populations vs. 5.6–6.0 per 1000 in type 2) [11, 28]. However, like the few previous studies in this area, this may be a descriptive rather than a statistical difference, or perhaps those with type 1 diabetes have a slightly more aggressive DFD pathophysiology; regardless further investigation is warranted [43].

### Strengths and limitations

The findings of this review should be interpreted cognisant of several limitations. First, although we searched databases using a robust published search strategy [16], we did not include grey literature and may have missed some government reports in particular. However, grey literature is rarely peer-reviewed and is difficult to identify with a reproducible search strategy, which risks compromising the robustness of this review. Second, we replaced the unvalidated quality assessment tool nominated in our published protocol [16] with a more appropriate validated quality assessment tool [17]. Last, the high heterogeneity of included publications identified prevented the pooling of outcomes using meta-analyses, and thus, this review is reliant on descriptive findings from rather diverse studies.

Conversely, a number of strengths should also be considered. First, it is the first review to comprehensively interrogate the literature for a range of DFD-related outcomes in Australia and provides the best evidence to date on the burden of DFD in Australia. Second, this review in the main adhered to published protocols, and where there was variation it was to improve the robustness of the published methodology. Third, we tested our search strategy using a validation set and used independent investigators to assess the eligibility of all records with very high agreement. Last, we used independent investigators who used a validated quality assessment tool developed for the purpose of assessing such epidemiology studies to quality assess all included papers.

With these strengths and limitations in mind, we recommend future robust epidemiology studies are urgently needed to confirm or refute the interpretations made from the heterogenous findings of this review. We strongly suggest such studies apply international validated standards for measuring DFD-related outcomes definitions within a nationally-representative population to better inform the national burden of DFD in Australia [4, 46]. However, until future robust studies return findings, the best available evidence suggests that Australia has a similar risk factor burden, an uncertain but perhaps low community DFD burden, but a high DFD-related hospitalisation and amputation burden compared to the rest of the world. We recommend clinicians, researchers and policymakers urgently investigate the national healthcare treatment of people with DFD to try and reduce what seems to be a very large burden of disease caused by the hospitalisation and amputation of Australians with DFD.

## Conclusion

Our review has identified comparably similar prevalence of risk factors, perhaps a low but uncertain prevalence of DFD, but high incidence of DFD-related hospitalisation and amputation in Australian populations. These findings may suggest that a low proportion of people with risk factors develop DFD, however, it is also possible that there is an underestimation of DFD prevalence in Australia in the few limited studies, given the high incidence of hospitalisation and amputation because of DFD. Furthermore, we found these high amputation rates were higher again in Indigenous and geographically remote populations with lower access to DFD treatment. We also found high heterogeneity between studies due to the use of different definitions for DFD outcomes, different epidemiology metrics and populations reported. Studies of nationally representative populations using valid outcome measures for DFD are needed to verify these findings.

## Supplementary Information

**Additional file 1: Table S1.** Search strings for PubMed and EMBASE. **Table S2.** Excluded papers (*N* = 15) during full-text assessment and reasons for exclusion. **Table S3.** Evidence table for all included publications that reported on risk factors for diabetes-related foot disease. **Table S4.** Evidence table for all included publications that reported on diabetes-related foot disease. **Table S5.** Evidence table for all included publications that reported on diabetes-related amputations. **Table S6.** Evidence table for all included publications that reported on aggregated risk factors or diabetes-related foot disease outcomes.

## Data Availability

Data sharing is not applicable to this article as no datasets were generated or analysed during the current study.

## Abbreviations

DFD: Diabetes-related foot disease
DFU: Diabetic foot ulcer
NS: Not stated
NT: Northern Territory
OECD: Organization for Economic Cooperation and Development
PAD: Peripheral arterial disease
PN: Peripheral neuropathy
PRISMA: Preferred Reporting Items for Systematic Review and Meta-Analyses
QA: Quality assessment
Qld: Queensland
Vic: Victoria
WA: Western Australia
