# Supplementary material for: Diabetes-related foot disease in Australia: a systematic review of the prevalence and incidence of risk factors, disease and amputation in Australian populations
Source: J Foot Ankle Res. 2021 Jan 19;14:8. doi: 10.1186/s13047-021-00447-x (PMC7816323; doi:10.1186/s13047-021-00447-x)
Supplement: Supplementary file 1 — Additional file 1: Table S1. Search strings for PubMed and EMBASE. Table S2. Excluded papers (N = 15) during full-text assessment and reasons for exclusion. Table S3. Evidence table for all included publications that reported on risk factors for diabetes-related foot disease. Table S4. Evidence table for all included publications that reported on diabetes-related foot disease. Table S5. Evidence table for all included publications that reported on diabetes-related amputations. Table S6. Evidence table for all included publications that reported on aggregated risk factors or diabetes-related foot disease outcomes. [file 13047_2021_447_MOESM1_ESM.docx]

**Supplementary materials for**

# **Diabetes-related foot disease in Australia: a systematic review of the prevalence and incidence of risk factors, disease and amputation in Australian populations**

**Table S1 Search strings for PubMed and EMBASE**

| PubMed | | EMBASE | |
| --- | --- | --- | --- |
| *#* | *Diabetic foot disease string* | *#* | *Diabetic foot disease string* |
| 1 | (“Ulcer”[Mesh] OR ulcer*[tw]) | 1 | ‘ulcer’/exp |
| **2** | (“Foot”[Mesh] OR foot[tw] OR feet[tw]) | **2** | ulcer:ab,ti |
| **3** | “Foot Ulcer”[Mesh] | **3** | #1 OR #2 |
| **4** | (“Foot Diseases”[Mesh] OR foot problem*[tw] OR foot disease*[tw]) | **4** | ‘foot’/exp |
| **5** | diabet*[tw] | **5** | (foot or feet):ab,ti |
| **6** | #1 AND #2 AND #5 | **6** | #4 OR #5 |
| **7** | #3 AND #5 | **7** | #3 AND #6 |
| **8** | #4 AND #5 | **8** | ‘foot ulcer’/exp |
| **9** | diabetic foot[tw] | **9** | ‘foot disease’/exp |
| **10** | diabetic feet[tw] | **10** | (foot disease* or foot problem*):ab,ti |
| **11** | #6 OR #7 OR #8 OR #9 OR #10 | **11** | diabet*:ab,ti |
|  |  | **12** | #7 AND #11 |
|  |  | **13** | #8 AND #11 |
|  |  | **14** | #9 AND #11 |
|  |  | **15** | #10 AND #11 |
|  |  | **16** | ‘diabetic foot’/exp |
|  |  | **17** | #12 OR #13 OR #14 OR #15 OR #16 |
|  | *Amputation string* |  | *Amputation string* |
| **12** | Amput* [tw] | **18** | ‘amput*’/exp |
| **13** | Diabet* [tw] | **19** | diabet*:ab,ti |
| **14** | #12 AND #13 | **20** | #18 AND #19 |
|  | Outcomes string |  | Outcomes string |
| **15** | Incidence | **21** | Incidence/exp |
| **16** | Prevalence | **22** | Prevalence/exp |
| **17** | #15 OR #16 | **23** | #21 OR #22 |
| **18** | Austral* | **24** | Austral*/exp |
| **19** | #17 AND #18 | **25** | #23 AND #24 |
|  | *Final search* |  | *Final search* |
| **20** | #11 OR #14 | **26** | #17 OR #20 |
| **21** | #19 AND #20 | **27** | #25 AND #26 |

**Table S2. Excluded papers (N=15) during full-text assessment and reasons for exclusion**

| **Author, year** | **Title** | **Reason for exclusion** |
| --- | --- | --- |
| Tapp, 2004 | Diabetes care in an Australian population: frequency of screening examinations for eye and foot complications of diabetes | Did not report outcomes of interest. |
| Schoen, 2014 | Diabetic foot disease in Indigenous people | Systematic review; The relevant publications identified in this review were included in our systematic review. |
| Dillon, 2017 | A systematic review describing incidence rate and prevalence of dysvascular partial foot amputation; how both have changed over time and compare to transtibial amputation | Did not differentiate people with and without diabetes |
| Kaminski, 2017 | Factors associated with foot ulceration and amputation in adults on dialysis: a cross-sectional observational study | Did not report outcomes of interest. |
| Kelly, 2017 | Major Lower Limb Amputation: Outcomes are Improving | Did not report outcomes of interest. |
| West, 2017 | Defining the gap: a systematic review of the difference in rates of diabetes-related foot complications in Aboriginal and Torres Strait Islander Australians and non-Indigenous Australians. | Systematic review; The relevant publications identified in this systematic review were included in our systematic review. |
| Behrendt, 2018 | International Variations in Amputation Practice: A VASCUNET Report. | Did not differentiate people with and without diabetes |
| Duke, 2018 | Diabetes mellitus after injury in burn and non-burned patients: A population based retrospective cohort study. | Did not report outcomes of interest. |
| Harding, 2018 | Global trends in diabetes complications: a review of current evidence | Systematic review; The relvant publications identified in this review were included in our systematic review. |
| Jeyaraman, 2019 | Mortality in patients with diabetic foot ulcer: a retrospective study of 513 cases from a single Centre in the Northern Territory of Australia. | Did not report a geographic catchment population of interest. |
| Lynar, 2019 | Risk factors for mortality in patients with diabetic foot infections: a prospective cohort study. | Did not report a geographic catchment population of interest |
| McCosker, 2019 | Chronic wounds in Australia: A systematic review of key epidemiological and clinical parameters | Systematic review; The relevant publications identified in this systematic review were included in our systematic review. |
| Wright, 2019 | Vascular surgery trends in Australia: 2001-2015: less open surgery, less limb loss and more endovascular intervention | Did not differentiate people with and without diabetes |
| Kaminski, 2019 | Risk factors for foot ulceration in adults with end-stage renal disease on dialysis: A prospective observational cohort study | Did not report a geographic catchment population of interest |
| Perrin, 2019 | The foot-health of people with diabetes in regional and rural Australia: baseline results from an observational cohort study | Did not report a specific geographic catchment population of interest |

**Table S3. Evidence table for all included publications that reported on risk factors for diabetes-related foot disease**

| **Reference** | **Study setting, period and design** | **Population definition, numbers** | **Age (SD) and male (%)** | **Foot outcomes included** | **Outcome definition** | **Prevalence or incidence** | **Outcome**  **numbers** | **Outcome (% or rate)** | **First or any occurrence** | **QA-total score** |
| --- | --- | --- | --- | --- | --- | --- | --- | --- | --- | --- |
| **Peripheral Neuropathy** | | | | | | | | | | |
| *Community dwelling* | |  |  |  |  |  |  |  |  |  |
| Baba,2014 | **Setting**: region-wide Freemantle, WA  **Design**: cohort study  **Period**: April 1993-June 1996 | T2 Diabetes,  **n**=1292 | **Age**: 64.0 (11.3)  **Male**: 48.6% | PN | Clinical examination: score >2/8 on the MNSI | Prevalence | 398 | 30.8% | Any | 7 |
| Baba,2015a | **Setting**: region-wide Freemantle, WA **Design**: cohort study  **Period**:2008-2011 | T2 Diabetes,  **n**=1509 | **Age**: 65.4(11.7)  **Male**: 51.8% | PN | As above | Prevalence | 878 | 58.2% | Any | 7 |
| Davis,2012 | **Setting**: region-wide Freemantle, WA **Design**: cohort study  **Period**:1993-1996 | T2 Aboriginal,  **n**=19 | **Age**: 51.2(11.4) **Male**: 36.8% | PN | As above | Prevalence | - | 38.9% | Any | 7 |
|  |  | T2 Anglo-Celt  **n**=796 | **Age**: 64.9(11.4) **Male**: 48.9% | PN | As above | Prevalence | - | 33.6% | Any | 7 |
|  | **Period**: 2008-2011 | T2 Aboriginal  **n**=106 | **Age:** 54.3(11.9)  **Male**: 34.9% | PN | As above | Prevalence | - | 48.5% | Any | 7 |
|  |  | T2 Anglo-Celt  **n**=796 | **Age**: 67.2(10.6)  **Male**: 50.9% | PN | As above | Prevalence | - | 63.3% | Any | 7 |
| Jia,2017 | **Setting**: state-wide Queensland  **Design**: prospective study  **Period**: Jan 2012- Dec 2014 | Diabetes patients with uninfected ulcer, **n**=853 | **Age**: 62.9 (12.8)  **Male**: 68.0% | PN | Clinically diagnosed as lack of protective sensation to a 10-gram monofilament on at least 2 of 3 plantar | Prevalence | 617 | 85.0% | Any | 9 |
| Tapp,2003 | **Setting**: nationwide Australia  **Design**: cross-sectional, population-based study  **Period**: 1999-2000 | T2 diabetes, **n**=821, including 398 Known diabetes (KDM) and 423 newly diagnosed diabetes (NDM) | **Age**: NS  **Male**: 52% | PN | Present if any two or more of the 4 scales were abnormal: NSS, NDS, PPT and postural blood pressure drop | Prevalence | 82 | 10.0% | Any | 9 |
| *Inpatients* | |  |  |  |  |  |  |  |  |  |
| Lazzarini,2017 | **Setting**: state-wide Queensland  **Design**: multi-site hospital-based observational study  **Period**: 2013 | Inpatients  **n**=733 | **Age:** 62.0(18.6) **Male:** 55.8% | PN | Clinical diagnosed as lack of protective sensation to a 10-gram monofilament on at least 2 of 3 plantar forefoot locations on at least one foot" | Point prevalence | 74 | 10.2% | Any | 7 |
|  |  | Diabetes inpatients  **n**=172 | **Age:** NS  **Male:** NS | PN | As above | Point prevalence | 74 | 43.3% | Any | 7 |
| **Peripheral Arterial Disease** | | | | | | | | | | |
| *Community dwelling* | |  |  |  |  |  |  |  |  |  |
| Baba,2014 | **Setting**: region-wide Freemantle, WA  **Design**: cohort study **Period**: April 1993-June 1996 | T2 Diabetes,  **n**=1292 | **Age**: 64.0 (11.3)  **Male**: 48.6% | PAD | Physical examination: ABI≤ 0.90 on either leg or a diabetes-related amputation was present | Prevalence | 377 | 29.2% | Any | 7 |
| Baba,2015a | **Setting**: region-wide Freemantle, WA  **Design**: cohort study  **Period**: April 1993-June 1996 | T2 Diabetes,  **n**=1292 | **Age**: 64.0 (11.3)  **Male**: 48.6% | Intermittent claudication | Self-reported, then ascertained by determining whether pain in the calves came on during walking, caused the patient to slow down or stop, and resolved with rest | Prevalence | 181 | 14.0% | Any | 6 |
|  |  |  |  | Peripheral arterial bypass/revascularisation | Hospital arterial bypass procedures from the WADLS | Prevalence | 17 | 1.3% | Any | 6 |
|  | **Period**:2008-2011 | T2 Diabetes,  **n**=1509 | **Age**: 65.4(11.7)  **Male**: 51.8% | PAD | As above | Prevalence | 341 | 22.6% | Any | 7 |
|  |  |  |  | Intermittent claudication | As above | Prevalence | 139 | 9.2% | Any | 6 |
|  |  |  |  | Peripheral arteria bypass/revascularisation | As above | Prevalence | 45 | 3.0% | Any | 6 |
| Davis,2012 | **Setting**: region-wide Freemantle, WA  **Design**: cohort study  **Period**:1993-1996 | T2 Aboriginal  **n**=19 | **Age**: 51.2(11.4)  **Male**: 36.8% | PAD | Clinical examination: ABI ≤ 0.90 on either leg or a diabetes-related amputation was present | Prevalence | - | 15.8% | Any | 7 |
|  |  | T2 Anglo-Celt  **n**=796 | **Age**: 64.9(11.4)  **Male**: 48.9% | PAD | As above | Prevalence | - | 29.7% | Any | 7 |
|  | **Period**: 2008-2011 | T2 Aboriginal  **n**=106 | **Age:** 54.3(11.9)  **Male**: 34.9% | PAD | As above | Prevalence | - | 30.7% | Any | 7 |
|  |  | T2 Anglo-Celt  **n**=796 | **Age**: 67.2(10.6)  **Male** 50.9% | PAD | As above | Prevalence | - | 21.5% | Any | 7 |
| Jia,2017 | **Setting**: state-wide Queensland  **Design**: prospective study  **Period**: Jan 2012- Dec 2014 | Diabetes patients with uninfected ulcer,  **n**=853 | **Age**: 62.9 (12.8)  **Male**: 68.0% | PAD | Clinical examination: toe systolic pressure <70 mmHg | Prevalence | 330 | 45.8% | Any | 9 |
| Tapp,2013 | **Setting**: nationwide Australia  **Design**: cross-sectional, population-based study  **Period**: 1999-2000 | T2 diabetes, **n**=821, including 398 KDM; 423 NDM | **Age:** NS  **Male**: 52% | PAD | Clinical examination: ABPI < 0.9, measured as ankle pressure/arm pressure on the right side, and ECQ requirements for claudication met | Prevalence | 84 | 10.3% | Any | 9 |
| *Inpatients* |  |  |  |  |  |  |  |  |  |  |
| Lazzarini,2016b | **Setting**: state-wide Queensland  **Design**: multi-site observational study  **Period**: 2013 | Diabetes inpatients  **n**=172 | **Age:** NS  **Male:** NS | critical PAD | Clinical examination: at least one absent foot pulse and a toe systolic pressure <30mmHg | Point prevalence | 12 | 7.0% | Any | 8 |
| Lazzarini,2017 | **Setting**: state-wide Queensland  **Design:** multi-site observational study  **Period**: 2013 | Inpatients  **n**=733 | **Age**: 62.0(18.6) **Male:** 55.8% | PAD | Clinical examination:"absence of at least one palpable foot pulse and a toe systolic pressure ≤70 mm Hg on at least one foot (mild: toe systolic pressure=51–70 mm Hg; moderate: toe systolic pressure=31–50 mm Hg; critical: toe systolic pressure=<30 mm Hg)" | Point prevalence | 60 | 8.2% | Any | 7 |
|  |  | Diabetes inpatients  **n**=172 | **Age:** NS  **Male:** NS | PAD | As above | Point prevalence | 60 | 35.1% | Any | 7 |
| O'Hara,1998 | **Setting**: state-wide Victoria,  **Design**: retrospective study  **Period**:1993-1995 | Hospital separations with diabetes  **n**=95091 | **Age:** NS  **Male**: 51.7% | PAD | Hospital coding: 4 digit ICD-9 code (not mentioned in the paper) | Prevalence | - | 2.7% | Any | 6 |
| **Previous foot ulcer** | |  |  |  |  |  |  |  |  | |
| *Community dwelling* | |  |  |  |  |  |  |  |  | |
| Baba,2015a | **Setting**: region-wide Fremantle, WA  **Design**: population-based cohort Study  **Period**: 1993-1996 | T2 Diabetes,  **n**=1296 | **Age**: 64.0(11.3)  **Male**: 48.6% | Previous foot ulcer | Self-reported foot ulcer "The patient was asked about prior foot ulceration" & hospital coding of hospitalisation "Details of prior hospitalisations accessed through the Western Australian Data Linkage System provided … ascertainment of … foot ulceration" | Prevalence | 6 | 0.5% | Any | 5 |
|  | **Period**: 2008-2011 | T2 Diabetes,  **n**=1509 | **Age**: 65.4(11.7)  **Male**: 51.8% | Previous foot ulcer | As above | Prevalence | 27 | 1.8% | Any | 5 |
| Jia,2017 | **Setting**: state-wide Queensland  **Design**: secondary analysis of a prospective study  **Period**: Jan 2012- Dec 2014 | Diabetes patients with uninfected ulcer, **n**=853 | **Age**: 62.9 (12.8)  **Male**: 68% | Previous foot ulcer | Self-reported in QHRFF | Prevalence | 595 | 69.8% | Any | 8 |
| Tapp,2013 | **Setting**: nationwide Australia  **Design**: cross-sectional, population-based study  **Period**: 1999-2000 | T2 diabetes, **n**=821, including n=398 KDM; n=423 NDM | **Age:** NS  **Male**: 52% | Previous foot ulcers | Self-reported in an interviewer-administered questionnaire | Prevalence | - | 2.1% | Any | 6 |
| *Inpatients* |  |  |  |  |  |  |  |  |  |  |
| Lazzarini,2017 | **Setting**: state-wide Queensland  **Design**: multi-site hospital-based observational study  **Period**: 2013 | Inpatients  **n**=733 | **Age**: 62.0(18.6) **Male**: 55.8% | Previous foot ulcer | Self-reported that they had a foot ulcer that healed, verified by a clinical examination | Point prevalence | 35 | 4.8% | Any | 7 |
|  |  | Diabetes inpatients  **n**=172 | **Age**: NS  **Male**: NS | Previous foot ulcer | As above | Point prevalence | 35 | 20.3% | Any | 7 |
| **Previous healed amputation** | |  |  |  |  |  |  |  |  |  |
| Jia,2017 | **Setting**: state-wide Queensland  **Design**: prospective study  **Period**: Jan 2012-Dec 2014 | Diabetes patients with uninfected ulcer,  **n**=852 | **Age**: 62.9 (12.8)  **Male**: 68% | Previous amputation | Clinical examination | Prevalence | 242 | 28.4% |  | 8 |
| Lazzarini,2017 | **Setting**: state-wide Queensland  **Design**: multi-site observational study  **Period**: 2013 | Inpatients  **n**=733 | **Age**: 62.0(18.6) **Male**: 55.8% | Previous amputation | Clinical examination | Point prevalence | 16 | 2.2% |  | 8 |
|  |  | Diabetes inpatients  **n**=172 | **Age**: NS  **Male**: NS | Previous amputation | As above | Point prevalence | 16 | 9.3% |  |  |
| **Foot deformity** | |  |  |  |  |  |  |  |  | |
| *Community dwelling* | |  |  |  |  |  |  |  |  | |
| Jia,2017 | **Setting**: state-wide Queensland, **Design**: prospective study  **Period**: 1 Jan 2012-31 Dec 2014 | Diabetes patients uninfected ulcer,  **n**=726 | **Age**: 62.9 (12.8)  **Male**: 68% | Foot deformity | Clinical diagnosis with a score of at least 3 points on a 6-point foot deformity score | Prevalence | 460 | 63.2% | Any | 9 |
| *Inpatients* |  |  |  |  |  |  |  |  |  |  |
| Lazzarini,2017 | **Setting**: state-wide Queensland  **Design**: multi-site hospital-based observational study  **Period**: 2013 | Inpatients  **n**=733 | **Age**: 62.0(18.6) **Male**: 55.8% | Foot deformity | Clinical examination:" having at least three of the following deformity characteristics on one foot: small muscle wastage, bony prominence, prominent metatarsal heads, hammer or claw toes, limited joint mobility, or Charcot deformity" | Point prevalence | 51 | 7.2% | Any | 7 |
|  |  | Diabetes inpatients  **n**=172 | **Age**: NS  **Male**: NS | Foot deformity | As above | Point prevalence | 51 | 30.5% | Any | 7 |

ABI: ankle brachial indices; ABPI: ankle brachial pressure index; ECQ: the Edinburgh Claudication questionnaire; ICD: International Classification of Disease; KDM: Known diabetes; MNSI: the Michigan Neuropathy Screening Instrument; NS: not stated; NDM: newly diagnosed diabetes; NDS: modified neuropathy disability score; NSS: neuropathy symptom score; PAD: peripheral arterial disease; PN: peripheral neuropathy PPT: pressure perception test; QHRFF: Queensland High Risk Foot Form; WADLS: Western Australian Data Linkage System

**Table S4**. **Evidence table for all included publications that reported on diabetes-related foot disease**

| **Reference** | **Study setting, period and design** | **Population definition, numbers,** | **Age (SD) and sex** | **Foot outcomes included** | **Firsts or any occurrence** | **Outcome definition** | **Prevalence or incidence** | **Outcome**  **numbers** | **Outcome % or rate (95% CI)** | **QA-total score** |
| --- | --- | --- | --- | --- | --- | --- | --- | --- | --- | --- |
| **Foot ulcer** | |  |  |  |  |  |  |  |  |  |
| *Community dwelling* | |  |  |  |  |  |  |  |  |  |
| Baba,2014 | **Setting**: region-wide Freemantle, WA  **Design**: cohort study  Baseline: April 1993-June 1996 | T2 Diabetes  **n**=1292 | **Age:** 64.0 (11.3)  **Male**: 48.6% | Foot ulcer | Any | Clinical examination located at or below the level of the malleoli | Prevalence | 16 | 1.2 (0.7-2.1) % | 5 |
|  | Follow-up for up to 17 years |  |  | Foot ulcer hospitalisation | First | Hospital coding: ICD-10-AM codes for primary diagnosis E1*.73 (diabetic foot ulcers) | Incidence | 79 | 5.2 (4.1-6.5) per 1000 PYs | 5 |
| Baba,2015b | **Setting**: region-wide Freemantle, WA  **Design**: cohort study  **Period**: 2008-2011 | T2 Diabetes  **n**=1509 | **Age**: 65.4 (11.7)  **Male**: 51.8% | Foot ulcer | Any | Clinical examination: "located at or below the level of the malleoli" | Prevalence | 22 | 1.5% | 5 |
| *Inpatients* |  |  |  |  |  |  |  |  |  |  |
| Lazzarini,2016a | **Setting**: state-wide Queensland  **Design**: multi-site hospital-based observational study  **Period**: 2013 | Inpatients  **n**=733 | **Age**: 62.0 (18.6)  **male**: 55.8% | Foot ulcer | Any | Clinical examination: an existing full-thickness wound beneath the ankle of primarily neuropathic, ischaemic, pressure injury origin or post-foot ulcer amputation site | Point prevalence | 26 | 3.5% | 8 |
|  |  | Diabetes inpatients  **n**=172 | **Age**: NS  **Male**: NS | Foot ulcer | Any | As above | Point prevalence | 26 | 15.1% | 8 |
| **Foot infection** | |  |  |  |  |  |  |  |  |  |
| *Community dwelling* | |  |  |  |  |  |  |  |  |  |
| Commons,2015 | **Setting**: region-wide Darwin, Northern Territory  **Design**: cohort study  **Period**: Sep 2012-Nov 2013 | General population  **n**=192,680 | **Age**: NS  **Male**: NS | Foot infection hospitalisation | Any | Hospital coding: IDSA classification on admission (uninfected, mild, moderate or severe). | Incidence | 245 | 79 per 100,000 PYs | 7 |
| Jia,2017 | **Setting**: state-wide Queensland  **Design**: secondary analysis of a prospective study  **Period**: 1 Jan 2012-31 Dec 2014 | T1 and T2 diabetes patients with uninfected ulcer  **n**=723 | **Age**: 62.9 (12.8)  **Male**: 68% | Foot infection | First | Clinical examination: defined according to the IWGDF classification system as at least two clinical signs or symptoms of infection in or around the DFU including purulence, erythema, pain, tenderness, warmth and/or induration | Incidence | 342 | 40.1% | 9 |
| *Inpatients* |  |  |  |  |  |  |  |  |  |  |
| Lazzarini,2016a | **Setting**: state-wide Queensland  **Design**: multi-site hospital-based observational study  **Period**: 2013 | Inpatients  **n**=733 | **Age**: 62.0 (18.6)  **Male**: 55.8% | Foot infection | Any | Clinical examination: at least two manifestations of inflammation (purulence, erythema, pain, tenderness, warmth or induration) | Point prevalence | 12 | 1.7% | 8 |
|  |  | Diabetes inpatients  **n**=172 | **Age**: NS  **Male**: NS | Foot infection | Any | As above | Point prevalence | 12 | 7.0% | 8 |

ICD-10-AM: International Classification of Disease, 10^th^ revision, Australian Modification codes; IDSA: the Infectious Diseases Society of America; IWGDF: the International Working Group on Diabetic Foot; PY: person years

**Table S5. Evidence table for all included publications that reported on diabetes-related amputations**

| **Reference** | **Study setting, period and design** | **Population definition, numbers** | **Age (SD) and sex** | **Foot outcomes included** | **Firsts or any occurrence** | **Outcome definition** | **Prevalence or incidence** | **Outcome**  **numbers** | **Outcome**  **(% or rate)** | **QA-total score** |
| --- | --- | --- | --- | --- | --- | --- | --- | --- | --- | --- |
| **Incidence** | |  |  |  |  |  |  |  |  |  |
| *Community dwelling* | |  |  |  |  |  |  |  |  |  |
| Clarke,2006 | **Setting**: state-wide Queensland  **Design**: case-control study  **Period**: 1 July 1995-30 June 1999 | Diabetes population, **n**=20538 | **Age**: 63.77(12.07)  **Male**: 50% | Total Amputation | Any | Hospital coding: ICD-9-CM Codes: 84.1X–81.4X | Incidence | **-** | 0.5%, 6.9/1,000 PY† | 7 |
| Commons,2015 | **Setting**: region-wide Darwin, Northern Territory  **Design**: prospective study **Period**: Sep 2012-Nov 2013 | General population  n=192,680 | **Age**: NS  **Male**: NS | Major amputation | Any | Surgical procedures: major amputations consisting of below and above knee amputations. | Incidence | 17 | 7.6/100,000 # | 7 |
|  |  |  |  | Minor amputation | Any | Surgical procedures: minor amputations were considered to be amputations below the ankle | Incidence | 54 | 24/100,000 # | 7 |
| Davis,2006 | **Setting**: region-wide Freemantle, WA  **Design**: cohort study  **Period**: 1993-2005 | T2 Diabetes,  **n**=1294 | **Age:** 64.0 (11.3)  **Male**: 48.6% | Total diabetes related LEA | Any | Hospital coding: ICD procedure codes, i.e. ICD-9-CM and ICD-10-AM, and confirmed be to for diabetic reasons. | Incidence | 48 | 6.0/1,000 PY | 5 |
|  |  |  |  | Total diabetes related LEA | First | Determined in patients without LEA at study entry by dividing the number of first-ever diabetes related LEAs by the patient-years of follow-up to first-ever LEA or death or 30 June 2005. | Incidence | 44 | 3.8/1,000 PY | 5 |
|  |  |  |  | Major diabetes related LEA | First | A major amputation was defined as through, or proximal to the tarsometatarsal joint. ICD procedure codes, i.e. ICD-9-CM and ICD-10-AM, and confirmed be to for diabetic reasons. | Incidence | 21 | 1.8/1,000 PY | 5 |
|  |  |  |  | Minor diabetes related LEA | First | A minor amputation was defined as one distal to this joint. ICD procedure codes, i.e. ICD-9-CM and ICD-10-AM, and confirmed be to for diabetic reasons. | Incidence | 27 | 2.3/1,000 PY | 5 |
| Dillon,2017 | **Setting**: nationwide Australia, **Design**: retrospective study **Period**:1 July 2007- 31 June 2012 | General population  n: NS | **Age**: NS  **Male**: NS | Amputation | Any | Hospital coding: ICD-10-AM-ACHI codes for amputation in presence of type 2 diabetes (E11) or type 1 diabetes (E10) from ANHM database | Incidence | 17,983 | 16.5/100,000^#^ | 8 |
| Kurowski,2015 | **Setting**: state-wide WA,  **Design**: retrospective study  **Period**: 2000-2010 | T1 and T2 diabetes  n=NS | **Age**: NS  **Male**: NS | Total amputation | Any | Hospital coding: ICD-10, with minor amputation defined as any amputation distal to the ankle joint (44338-00,44358-00, 44364-00, 44364-01, 90557-00) and major amputation as through or proximal to the ankle joint (44361-00, 44361-01, 44367-00, 44367-01, 44367-02). | Incidence | 3763 (T1: 364, T2: 3399) | Subgroups: T1:7.2/1,000  T2:5.6/1,000 | 7 |
|  |  |  |  | Major amputation | Any | As above | Incidence | (T1:109, T2:1082) | Subgroups: T1:2.4/1,000  T2:1.8/1,000 | 7 |
|  |  |  |  | Minor amputation | Any | As above | Incidence | (T1:255, T2: 2317) | Subgroups: T1:4.8/1,000  T2:3.9/1,000 | 7 |
|  |  |  |  | Initial total amputation | First | Identified as initial if the patient had a 15-year amputation-free history | Incidence | 2156 (T1:179  T2:1977) | Subgroups:  T1: 3.7/1,000  T2:3.4/1,000 | 7 |
|  |  |  |  | Initial Major amputation | First | Identified as initial if the patient had a 15-year amputation-free history, with major amputation defined as above | Incidence | (T1:44, T2: 698) | Subgroups: T1: 1.1/1,000  T2:1.0/1,000 | 7 |
|  |  |  |  | Initial Minor amputation | First | Identified as initial if the patient had a 15-year amputation-free history, with minor amputation defined as above | Incidence | (T1:135, T2:1367) | Subgroups: T1: 2.6/1,000  T2:2.4/1,000 | 7 |
|  |  |  |  | Recurrent Total amputation | Recurrent | Other amputation not identified as initial | Incidence | 1607 (T1:185  T2:1422) | Subgroups:  T1: 96.9/ 1,000  T2:354.4/1,000 | 7 |
|  |  |  |  | Recurrent major amputation | Recurrent | Major amputation not identified as initial | Incidence | (T1:65, T2:472) | Subgroups: T1:45.0/1,000  T2:111.6/1,000 | 7 |
|  |  |  |  | Recurrent minor amputation | Recurrent | Minor amputation not identified as initial | Incidence | (T1:120, T2:950) | Subgroups: T1:59.1/1,000  T2:240.7/1,000 | 7 |
| Lazzarini,2015 | **Setting**: state-wide Queensland, **Design** retrospective study  **Period**: 1 January 2005-31 December 2010 | 1.Diabetes population:  n=846,967  2.General population:  n=24,990,524 | **Age**: NS  **Male**: NS | Total Amputation | Any | Hospital coding: any lower extremity amputation procedural code identified in the admitted patient | Incidence | 4443 | 5.2/1,000 in diabetes population;  17.8/100,000 in general population | 7 |
|  |  |  | **Age**: NS  **Male**: NS | Minor Amputation | Any | Hospital coding: amputation procedures distal to the ankle | Incidence | 3009 | 3.5/1,000 in diabetes population;  12.0/100,000 in general population | 7 |
|  |  |  |  | Major Amputation | Any | Hospital coding: amputation procedures through or proximal to the ankle | Incidence | 1434 | 1.7/1,000 in diabetes population;  5.8/100,000 in general population | 7 |
| Norman,2010 | **Setting**: state-wide WA,  **Design**: retrospective study  **Period**: 2000-2008 | Indigenous population  n: NS | **Age**: NS  **Male**: NS | Minor amputation | Any | Hospital coding: toe or foot amputations using ICD-10-AM codes | Incidence (ASR) | - | **age group**:25-49:  46.4/100,000  **age group**: ≥50: 185/100,000 | 6 |
|  |  |  |  | Major amputation | Any | Hospital coding: amputations below or above the knee using ICD-10-AM codes | Incidence  (ASR) | - | **age group**: 25-49: 15.0/100,000**age group**:25-49: 0.15/1,000;  **age group**: ≥50: 76.8/100,000 | 6 |
|  |  | Non-Indigenous population  n: NS | **Age**: NS  **Male**: NS | Minor amputation | Any | Hospital coding: toe or foot amputations using ICD-10-AM codes | Incidence (ASR) | - | **age group**:25-49:  1.7/100,000;  **age group**: ≥50:  28.9/100,000. | 6 |
|  |  |  |  | Major amputation | Any | Hospital coding: amputations below or above the knee using ICD-10-AM codes | Incidence  (ASR) | - | **age group**:25-49:  0.4/100,000;  **age group**: ≥50: 13.1/100,000. | 6 |
| O'Rourke,2012 | **Setting**: region-wide Far North Queensland, **Design**: retrospective study **Period**: 1999-2008 | General population  n: 262,000 | **Age**: NS  **Male**: NS | Major amputation | Any | Hospital coding: patients with a primary or secondary diagnosis of diabetes mellitus (specified as E10–E14) and a major limb amputation (specified as 4437000, 4437300, 4436700, 4436701 and 4436702) | Incidence (ASR) | 16 | 9.3  /100,000 per year | 6 |
| Payne,2000 | **Setting**: Australia, **Design**: retrospective study  **Period**: 1995-1998 | General population  n: NS | **Age**: NS  **Male**: NS | Total Amputation | Any | Hospital coding: ICD-9 procedure codes 84.10 to 84.19 (amputations of the lower extremity) and diagnosis codes 250.0 to 250.9 (indicating diabetes and its complications) as the principal or secondary diagnoses from ANHM database | Incidence | 7887 | age-and sex-standardized rates: 14.0/100,000 | 8 |
| **Prevalence** | |  |  |  |  |  |  |  |  |  |
| Lazzarini,2016b | **Setting**: state-wide Queensland  **Design**: multi-site observational study  **Period**: 2013 | Diabetes inpatients  **n**=172 | **Age**: NS  **Male**: NS | Amputation procedure | Any | Clinical examination | Point prevalence | 10 | 5.8% | 8 |
| O'Hara,1998 | **Setting**: state-wide Victoria, **Design**: retrospective study **Period**: 1993-1995 | Hospital separation with diabetes,  **n**=95091 | **Age:** NS  **Male**: 51.7% | Amputation procedure | Any | ICD-9 procedure code 841 | Prevalence | 1281 | 1.4% | 6 |

ASR: age-standardised rate; ANHM: Australian National Hospital Morbidity Database; ICD-9-CM: International Classification of Disease, 9^th^ revision, Clinical Modification codes; ICD-10-AM: International Classification of Disease, 10^th^ revision, Australian Modification codes; ICD-10-AM-ACHI: International Classification of Disease, 10^th^ revision, Australian Modification Australian Classification of Health Interventions; PY: person years

† Rate calculated based on the article; ^#^ Incidence was calculated by dividing the numerator of diabetes related-amputations by the denominator of population for the period as reported in the paper. The numerator of diabetes related-amputation was calculated as 17,983 for the period = 1,596 type 1 + 16,387 type 2 amputations. The denominator was calculated as 108,969,135 for the period = 35,306 total amputations / (32.4/100,000 total incidence reported).

**Table S6. Evidence table for all included publications that reported on aggregated risk factors or diabetes-related foot disease outcomes**

| Reference | Study setting, period and design | Population definition, numbers | Age (SD) and male | Foot outcomes included | First or any occurrence | Definition | Prevalence or incidence | Outcome  numbers | Outcome (% or rate) | QA-total score |
| --- | --- | --- | --- | --- | --- | --- | --- | --- | --- | --- |
| *Community-dwelling* | | | | | | | | | | |
| Ewald,2001 | **Setting**: region-wide Central Australia,  **Design**: secondary data analysis  **Period**: 1 Jan 1992-31 Dec 1997 | General population | **Age**: NS  **Male**: NS | Foot complication hospitalisation | Any | Hospital coding: ICD-9-CM codes for lower limb ulcer, lower limb amputation, lower limb infection, debridement, peripheral neuropathy or peripheral vascular disease in any comorbidity or procedure fields. | Incidence | - | 98-285/100,000 | 5 |
| Lazzarini,2015 | **Setting**: state-wide Queensland, **Design**: retrospective study  **Period**: January 2005- December 2010 | Community-dwelling population with diabetes  **n**=846,967 | **Age**: NS  **Male**: NS | Hospitalisation for diabetes related foot complication | Any | Hospital coding: ICD diagnosis codes for a foot related complication including neuropathy, PAD, foot ulcers, infections, Charcot and amputation procedures) | Incidence | 24,917 | 2005: 36.6/1,000;  2010:  20.9 /1,000 ^ | 7 |
| *Inpatients* |  |  |  |  |  |  |  |  |  |  |
| Lazzarini,2016a | **Setting**: state-wide Queensland  **Design**: hospital-based multi-site observational study  **Period**: 2013 | Diabetes inpatients  **n**=172 | **Age**: NS  **Male**: NS | Admission for foot related condition (primary reason) | Any | Self-reported identification of a foot-related condition as primary or secondary reason for admission | Prevalence | 15 | 8.7% | 7 |
|  |  |  |  | Admission for foot related condition (secondary reason) | Any | Self-reported identification of a foot-related condition as primary or secondary reason for admission | Prevalence | 19 | 11.0% | 7 |
|  |  |  |  |  |  |  |  |  |  |  |
| Lazzarini,2017 | **Setting**: state-wide Queensland  **Design**: hospital-based multi-site observational study  **Period**: 2013 | Diabetes inpatients  **n**=172 | **Age**: NS  **Male**: NS | Foot complications | Any | defined as participants with at least one foot complication including previous foot ulcer, previous amputation, PN, PAD and foot deformity | Prevalence | 112 | 65.5% | 7 |
|  |  |  |  | Multiple foot complications | Any | defined as participants with two or more foot complication including previous foot ulcer, previous amputation, PN, PAD and foot deformity | Prevalence | 66 | 38.6% | 7 |

^Annual incidence was reported from 2005 to 2010, and the incidence of 2005 and 2010 is presented in the table.
